# The Maltine Company’s Prizes

**Published:** 1902-02

**Authors:** Charles C. Heuman

**Affiliations:** Secretary Maltine Company, Borough of Brooklyn, New York City


### CORRESPONDENCE.

### The Maltine Company's Prizes.

*Editor Buffalo Medical Journal:*

*Sir.*—Supplementing the new advertisement sent to you on the 8th inst., I enclose herewith a circular which is now being mailed to every physician in the United States. This, I think, is one of the most unique enterprises in which our company has ever engaged, and that much interest and comment will follow its promulgation.

The scheme has been under consideration for some time, a number of the leading practitioners in various parts of the country have been consulted concerning it, and cordial approval was signified in every instance.

The prizes are large, the judges of high standing, and the conditions governing the competition are such as to enable the most ethical of men to compete without compromising themselves. You will notice that the three judges are not only men of the first rank, but represent different sections of the country and different branches of the profession, Dr. Lewis being a surgeon and editor of one of the leading medical journals, Dr. Reed, a gynecologist and former president of the American Medical Association, and Dr. Rhodes, a purely medical man.

Then, again, the subject is such a broad one that every inteligent practitioner, whether he makes a specialty of surgery, pediatrics, gynecology or internal medicine, or is simply an all around family doctor, can write upon it.

We depend very largely upon our friends in medical journalism to bring this matter properly and effectively before the medical profession, and I feel sure your coöperation and support can be depended upon.

CHARLES C. HEUMAN,
*Secretary Maltine Company.*

BOROUGH OF BROOKLYN, NEW YORK CITY, January 10, 1902.

